# Additive Manufacturing and Characterization of Continuous Nettle Fiber-Reinforced PLA Composites

**DOI:** 10.3390/polym17172388

**Published:** 2025-08-31

**Authors:** Ahmet Cagri Kilinc

**Affiliations:** Department of Mechanical Engineering, Osmaniye Korkut Ata University, Osmaniye 80010, Turkey; ahmetcagrikilinc@osmaniye.edu.tr

**Keywords:** nettle fiber, continuous natural fiber, 3D printing, FDM, mechanical properties

## Abstract

Continuous nettle fiber-reinforced PLA composites were fabricated using a custom-designed fused deposition modeling (FDM) 3D printer equipped with an in-nozzle fiber impregnation system. The influence of hatch spacing and layer thickness on fiber volume fraction, tensile strength, and fracture surface morphology was systematically examined. Fiber content increased from 7.94 vol.% to 12.21 vol.% when hatch spacing was reduced from 1.0 mm to 0.6 mm at a constant 0.4 mm layer thickness, and from 12.21 vol.% to 24.43 vol.% when layer thickness was decreased from 0.4 mm to 0.2 mm at a fixed 0.6 mm hatch spacing. When compared to neat PLA, tensile strength was improved by 18.69% for the configuration of 1_04 and 75.83% for the configuration of 06_02. SEM analysis revealed orderly fiber deposition in all samples, with 3D-printing-induced voids and fiber pull-out observed on fracture surfaces. Reduced hatch spacing and layer thickness resulted in denser fiber packing, consistent with mechanical performance trends. The results highlight the strong influence of printing parameters on the microstructural and mechanical behavior of continuous natural fiber composites produced by FDM.

## 1. Introduction

In the face of growing environmental challenges and an urgent global shift towards sustainability, the development of eco-friendly and renewable materials has become a focal point of materials science research [1]. Natural fiber-reinforced polymer composites have emerged as highly promising alternatives to traditional synthetic fiber composites due to their numerous environmental and functional benefits [2]. These natural fibers, derived from abundant plant sources such as flax, hemp, jute, and nettle (Urtica dioica), combine renewability, biodegradability, and low density with favorable mechanical properties [3,4]. Such attributes position them as attractive reinforcements in polymer matrices, especially when paired with biodegradable polymers like polylactic acid (PLA), thereby enabling the fabrication of composites that are not only high-performing but also environmentally responsible [5]. This paradigm aligns closely with circular economy by reducing dependence on non-renewable resources.

Among various natural fibers, nettle fiber has garnered increasing attention in recent years due to its unique combination of mechanical robustness and sustainable cultivation characteristics [6]. Nettle is a fast-growing herbaceous plant that requires minimal agrochemical inputs, including fertilizers and pesticides, making it an ecologically preferable fiber source compared to more intensively farmed crops [3]. Its fibers exhibit tensile strength and stiffness values comparable to well-established natural fibers like flax and hemp, while its cultivation supports local economies and promotes biodiversity [7,8]. These factors collectively render nettle fiber a highly promising reinforcement material for bio-based composites, capable of satisfying both performance and sustainability criteria.

The advantages of natural fibers as composite reinforcements extend beyond their environmental credentials. Their low density significantly contributes to the development of lightweight composites, which is especially critical in automotive, aerospace, and construction sectors where weight reduction translates directly to enhanced fuel efficiency and lower emissions. Furthermore, natural fibers are cost-effective relative to synthetic fibers such as glass or carbon, facilitating economic manufacturing of composite components without compromising sustainability goals [9,10].

Despite these benefits, the integration of natural fibers into polymer matrices is not without challenges. One of the significant challenges is related to fiber length: the majority of natural fiber composites currently utilize short or discontinuous fibers due to processing constraints, which inherently limit stress transfer efficiency and mechanical reinforcement [11,12]. As a result, these composites typically do not achieve mechanical properties comparable to those reinforced with continuous fibers, restricting their use in load-bearing or structural applications [13].

Continuous fiber reinforcement has emerged as a promising strategy to overcome these limitations. Continuous fibers provide uninterrupted load paths within the composite, facilitating more effective stress transfer from the matrix to the reinforcement [14]. This translates into substantially improved mechanical properties, including higher tensile strength and stiffness. While the advantages of continuous synthetic fiber composites are well-documented, the potential for continuous natural fiber reinforcements remains relatively underexploited, particularly in the context of emerging fabrication technologies. Additive manufacturing, notably FDM, presents an innovative platform to integrate continuous fibers into complex geometries with high precision and customization [14,15]. Among the range of additive manufacturing (AM) technologies not only utilized for neat polymer processing but also for metal, ceramic, and composite production, including selective laser melting (SLM), digital light processing (DLP), binder jetting (BJ), and fused deposition modeling (FDM) [16], FDM stands out as one of the most accessible and cost-effective methods [17,18,19,20,21,22,23]. The inherent layer-by-layer deposition process of FDM, combined with continuous fiber impregnation, allows for tailoring fiber orientation, volume fraction, and distribution within the printed part, enabling control over composite microstructure and mechanical behavior [24].

In this context, this research focuses on the production and characterization of continuous nettle fiber-reinforced PLA composites. The study systematically examines the influence of key printing parameters, namely hatch spacing and layer thickness, on the fiber volume fraction and mechanical properties of the composites.

By exploring how these parameters affect the microstructural and mechanical properties of the printed composites, this study aims to bridge the gap between the environmental advantages of natural fiber reinforcements and the mechanical demands of advanced manufacturing applications. Through this approach, the study contributes to expanding the applicability of sustainable materials in additive manufacturing.

## 2. Materials and Methods

The matrix material employed in this study was a commercially available PLA filament with a diameter of 1.75 mm, supplied by ESUN (Shenzhen Esun Industrial Co., Ltd., Shenzhen, China). PLA, a biodegradable thermoplastic aliphatic polyester derived from renewable resources such as corn starch or sugarcane, was selected due to its favorable mechanical properties, low environmental footprint, and compatibility with additive manufacturing processes [25]. The reinforcing phase consisted of continuous nettle fibers, which were sourced in yarn form and subsequently prepared for composite fabrication. Nettle fibers were chosen as the reinforcement owing to their high specific strength, stiffness, and renewability, combined with their low density, making them an attractive alternative to conventional synthetic fibers for sustainable composite production. The chemical composition of nettle fibers and properties of PLA filament are given in Table 1.

Both the PLA matrix and the nettle yarn were stored under controlled laboratory conditions to minimize moisture uptake prior to fabrication, as excess humidity can adversely affect the processing quality and interfacial bonding. The PLA filament was maintained in sealed, desiccated container, while the nettle yarns were dried in an oven at 60 °C for a period of 24 h to reduce their moisture content. The combination of these materials was selected to produce a bio-based composite with a balance of mechanical performance, reduced environmental impact, and suitability for processing using FDM techniques.

**Figure 1:**
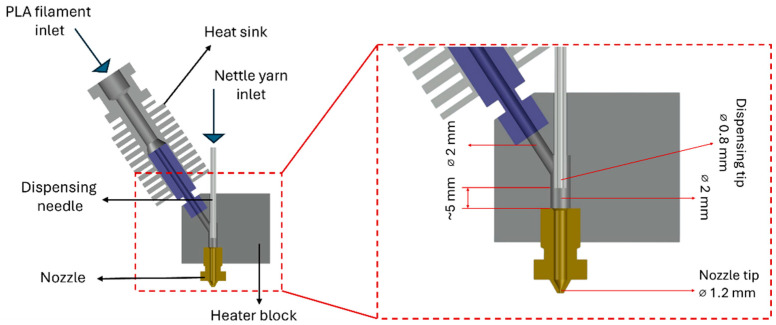
Continuous fiber extruder.

A custom-made extrusion head was developed for the 3D printing of continuous natural fiber-reinforced PLA composites using the nozzle impregnation method. A schematic representation of the continuous fiber extruder is shown in Figure 1. In this configuration, the thermoplastic matrix and the continuous fiber were fed from separate inlets, converging in an aluminum heating block where the molten PLA impregnated the fiber prior to extrusion. The design concept was adapted from the hot-end configurations reported by Tian et al. [27] and Ibrahim et al. [28] for continuous fiber-reinforced composite fabrication. Both feeding channels were integrated into the heating block to promote impregnation, and the resulting composite filament was deposited through the nozzle.

**Figure 2:**
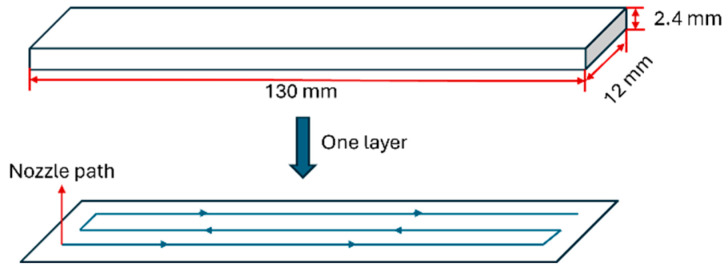
Mechanical test specimen dimensions and continuous printing path representation.

The printing process was carried out using a custom-designed Cartesian-type FDM 3D printer, in which the printhead moves along the X and Z axes, while the build plate travels along the Y axis. For unidirectional composite fabrication with a fiber orientation of 0°, a printing path strategy with continuous nozzle-head movement was performed. Specimens with dimensions of 130 mm × 12 mm × 2.4 mm were 3D printed for mechanical tests. Tensile tests were performed according to ASTM D3039 under ambient conditions with a cross-head speed of 2 mm/min. Three specimens were tested for each printing configuration. Schematic representations of mechanical test specimens and the single layer with a continuous printing path are shown in Figure 2.

Specimens were 3D printed by using various layer thicknesses and hatch spacing for investigation of the effect of nettle fiber reinforcement on the properties of the composites. Detailed printing parameters are given in Table 2. The specimen labels according to printing parameters are given in Table 3.

The determination of the fiber volume fraction in the composites was adapted from the methodology reported by Waterbury and Drzal [29], which involves determining the ratio of fiber area to the total cross-sectional area of the sample. This approach assumes that the cross-sectional geometry remains constant along the entire length of the specimen; therefore, the area fraction is taken to be equivalent to the volume fraction [30]. Using this method, the fiber volume fractions for composites fabricated via 3D printing under varying hatch spacing and layer thickness were calculated as follows:

(1) $$V_{f}=\frac{N_{t}\times A_{t}}{A_{s}},$$

The fiber volume fraction, *V_f_*, is defined as the ratio of the total cross-sectional area of the yarn—calculated by multiplying the number of yarn in the cross section, *N_t_*, by the cross-sectional area of a single yarn, *A_t_*—to the total cross-sectional area of the specimen, *A_s_*.

The nettle fiber was subjected to a series of physicochemical and morphological characterizations. X-ray diffraction (XRD, Rigaku D/MAX) was employed to determine the crystalline structure and phase composition of the fibers. The crystallinity index (CI) of the fibers was determined according to the empirical formula proposed by Segal et al. [31] as follows:

(2) $$CI(\%)=\frac{I_{200}-I_{AM}}{I_{200}},$$

*I*_200_: Maximum intensity of the peak corresponding to the plane in the sample with Miller indices (200). *I_AM_*: Intensity of the amorphous region, which is the valley located between the two peaks [9].

Fourier-transform infrared spectroscopy (FT-IR, Perkin Elmer Spectrum BX) was used to identify the functional groups present in the fiber. The spectrum was recorded in the range of 650–4000 cm^−1^ with a resolution of 2 cm^−1^.

The thermal behavior of nettle yarn, composite specimens, and neat PLA was investigated by means of thermogravimetric analysis (TGA). The measurements were conducted under a nitrogen atmosphere within a temperature range of 30–600 °C, applying a constant heating rate of 10 °C/min.

The surface morphology and microstructural features of the fibers were examined using scanning electron microscopy (SEM). For the composite specimens, the fracture surfaces of continuous nettle fiber-reinforced PLA composites after tensile testing were analyzed by SEM to elucidate the failure mechanisms and fiber–matrix interfacial behavior. All samples were Au-Pd coated by using a sputter-coating device for 45 s before SEM examinations.

## 3. Results and Discussion

### 3.1. Fiber Characterization

**Figure 3:**
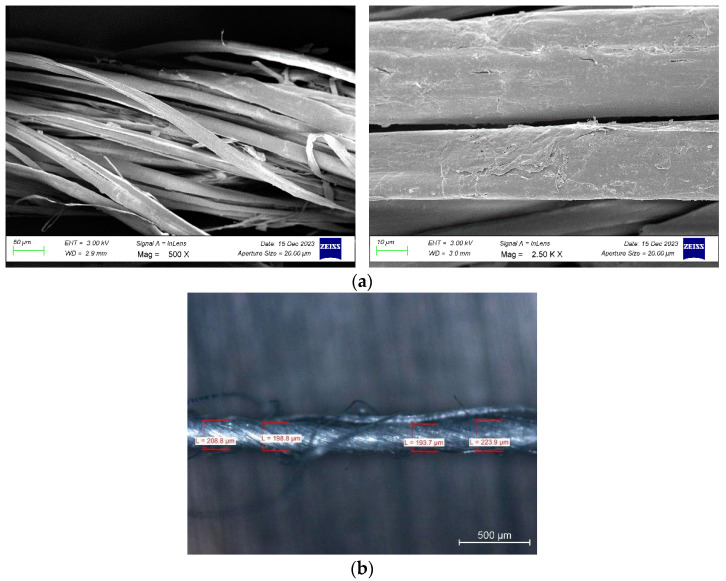
(**a**) SEM images; (**b**) optical microscope image of nettle fiber yarn.

SEM images of the nettle fibers are presented in Figure 3a. Measurements revealed that the fiber diameters varied within the range of 9.12 µm to 35.41 µm with a relatively smooth surface, indicating a notable diversity in fiber thickness. The SEM micrographs clearly display the twisted morphology. In addition, the optical microscope images of the yarns are shown in Figure 3b. The average yarn diameter was determined to be 206.9 ± 13.3 µm, and the surface texture highlights the bundled arrangement of individual fibers within the yarn structure.

**Figure 4:**
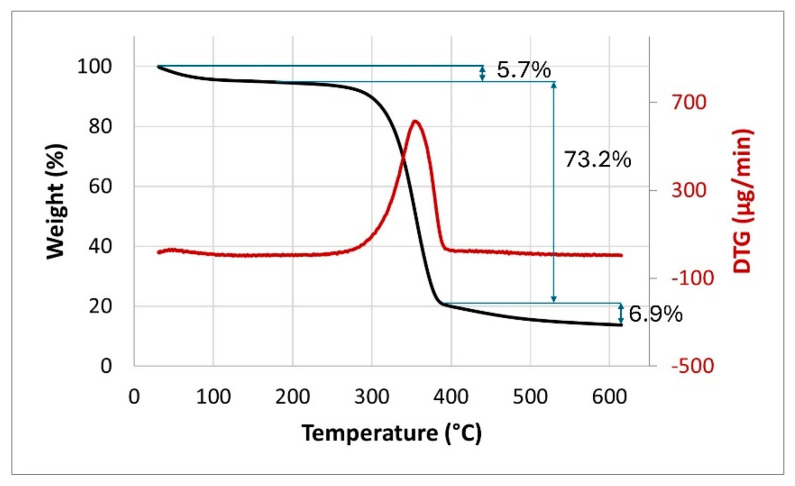
TGA and DTG curves of nettle fiber.

TG and DTG curves of the nettle fiber are shown in Figure 4. The thermogravimetric (TG) curve of the nettle fibers demonstrates a multistage degradation profile typical of lignocellulosic materials. An initial slight weight loss below approximately 100 °C (5.7%) was attributed to the evaporation of physically adsorbed moisture and volatile compounds [1]. The main degradation event occurs between 269 and 389 °C, where a sharp mass loss is observed (73.2%). This stage corresponds to the thermal decomposition of hemicellulose and cellulose, which are the dominant components of the fiber structure [9]. The maximum degradation rate (T_max_) appears around 353 °C, while the onset of major decomposition (T_onset_) is detected at approximately 269 °C. Beyond 389 °C, the curve levels off, indicating the formation of a relatively stable char residue, which remains up to 600 °C. The presence of this residual mass suggests the contribution of lignin, which degrades slowly over a wider temperature range and enhances the char yield [10]. A total of 6.9% weight loss was observed between 389 and 600 °C, and beyond this temperature a resultant char yield of 14.2% was observed. Overall, the TG analysis confirms the lower thermal stability of nettle fibers compared to PLA, consistent with the nature of natural lignocellulosic fibers. The DTG curve of nettle fibers exhibits a typical multi-step degradation pattern. A minor peak below 100 °C corresponds to the evaporation of absorbed water and low-molecular-weight volatiles. The major degradation event occurs between approximately 269 and 389 °C, where a sharp and intense peak is observed, with the maximum degradation rate (T_max_) centered around 353 °C [2]. This stage is primarily associated with the decomposition of hemicellulose and cellulose, which constitute the main structural components of the fiber. Beyond 389 °C, a low and broad signal is detected, attributed to the gradual degradation of lignin, which decomposes over an extended temperature range. The persistence of this signal indicates the contribution of lignin to char residue formation, consistent with the TG results.

**Figure 5:**
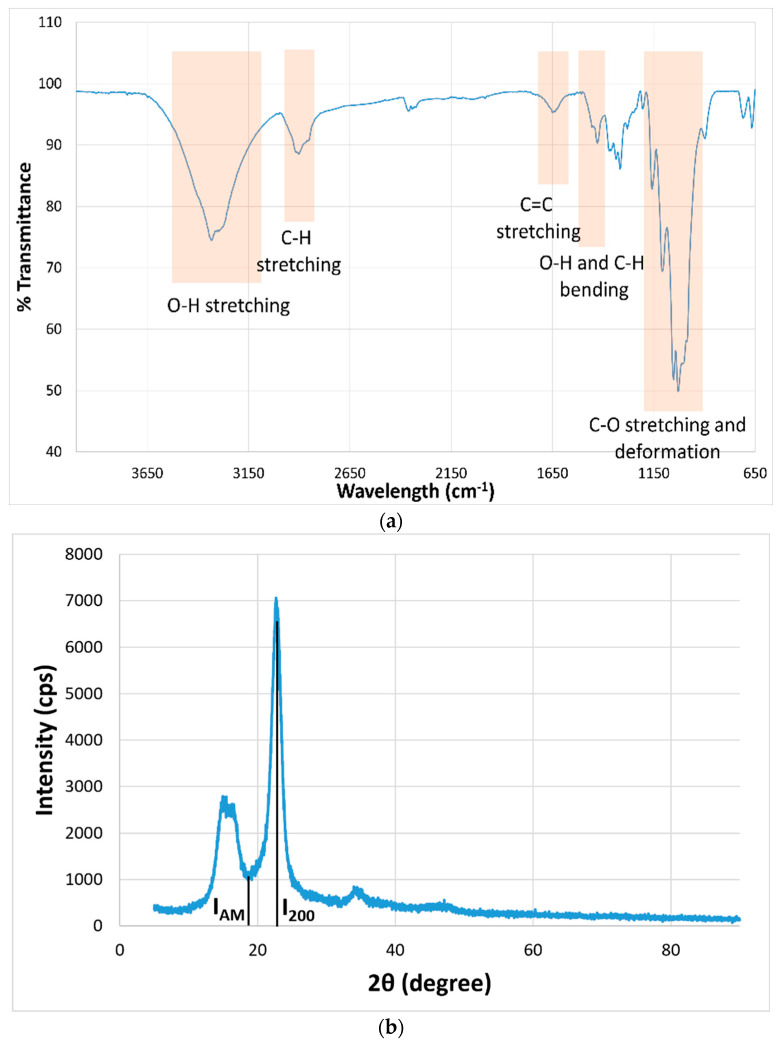
(**a**) FT-IR spectrum; (**b**) XRD pattern of nettle fiber yarn.

The FT-IR spectrum of the fiber is shown in Figure 5a. The FT-IR spectrum of nettle fiber exhibits several characteristic absorption bands corresponding to the functional groups present in its lignocellulosic structure. A broad and intense band observed in the region of approximately 3350–3300 cm^−1^ is attributed to the O–H stretching vibrations of hydroxyl groups, which are abundant in cellulose, hemicellulose, and bound water molecules [32]. The absorption peak near 2900 cm^−1^ corresponds to C–H stretching vibrations of aliphatic –CH_2_ groups in polysaccharide chains [33]. The band appearing around 1630–1640 cm^−1^ is generally linked to the H–O–H bending vibration of water in lignin or cellulose [34,35,36]. Additional peaks in the range of 1150–1160 cm^−1^ indicate asymmetric stretching of C–O–C glycosidic linkages, characteristic of polysaccharide backbones [37,38]. The presence and relative intensity of these bands confirm the typical chemical composition of nettle fibers, dominated by cellulose with contributions from hemicellulose, lignin, and minor extractives.

The XRD pattern of the fiber is shown in Figure 5b. X-ray diffraction (XRD) analysis of the nettle yarn revealed a distinct crystalline structure, as evidenced by the well-defined diffraction peaks in the obtained pattern. X-ray diffraction analysis revealed two sharp peaks at 2θ angles of 16.30° and 22.72°, corresponding to the [110] and [200] planes, along with a broad peak at 34.68°, attributed to the [004] plane [39,40]. Using the Segal empirical approach, the degree of crystallinity was calculated based on the intensity ratio between the characteristic crystalline peak and the amorphous background. The crystallinity index was determined as 85.8%, indicating a highly ordered arrangement of cellulose chains within the yarn. Such a high crystallinity value suggests enhanced stiffness, dimensional stability, and potential improvements in mechanical performance, which can be attributed to the strong hydrogen bonding and dense packing of cellulose microfibrils. This pronounced crystalline order is consistent with the inherent structural characteristics of lignocellulosic bast fibers and can significantly influence the thermal behavior, moisture absorption, and interfacial adhesion properties when used as reinforcement in polymer composites [2].

### 3.2. Composite Characterization

**Figure 6:**
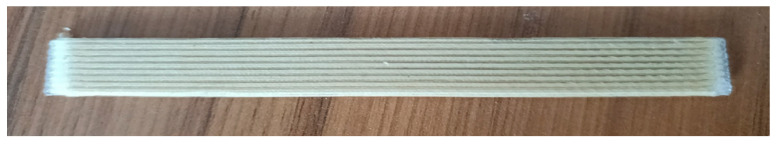
The image of 3D-printed continuous nettle fiber-reinforced PLA composite.

An image of the 3D-printed continuous nettle fiber-reinforced PLA composite is shown in Figure 6. From a macroscopic perspective, the specimens exhibit the characteristic architecture of a typical continuous fiber-reinforced composite. The continuous reinforcement phase is clearly discernible within the transparent PLA matrix, allowing the fiber distribution to be readily observed. At the terminal regions of the samples, slight fiber retractions are noticeable, which are likely attributable to the tensile load applied to the nettle fibers during printing. Overall, the composite displays a uniform and homogeneous morphology, indicative of effective impregnation and consolidation. Notably, no signs of fiber misalignment were detected, suggesting that the reinforcement was maintained in an optimal orientation throughout fabrication.

The fiber reinforcement ratios obtained for the composites produced under varying layer thickness and hatch spacing parameters are presented in Table 4. Analysis of the fiber volume fractions revealed a clear dependence of reinforcement content on printing parameters. For a constant layer thickness of 0.4 mm, decreasing the deposition hatch spacing resulted in an increase in fiber content from 7.94% to 12.21%. Similarly, when the hatch spacing was held constant at 0.6 mm, reducing the layer thickness from 0.4 mm to 0.2 mm led to a marked increase in fiber content from 12.21% to 24.43%. This trend can be attributed to the fact that reduced layer thickness and narrower hatch spacing necessitate a greater number of deposited lines per unit area, which inherently increases the number of fiber insertions within that area [41,42,43]. Consequently, the overall reinforcement content rises, enhancing the potential for improved mechanical performance through higher fiber-to-matrix ratios.

**Figure 7:**
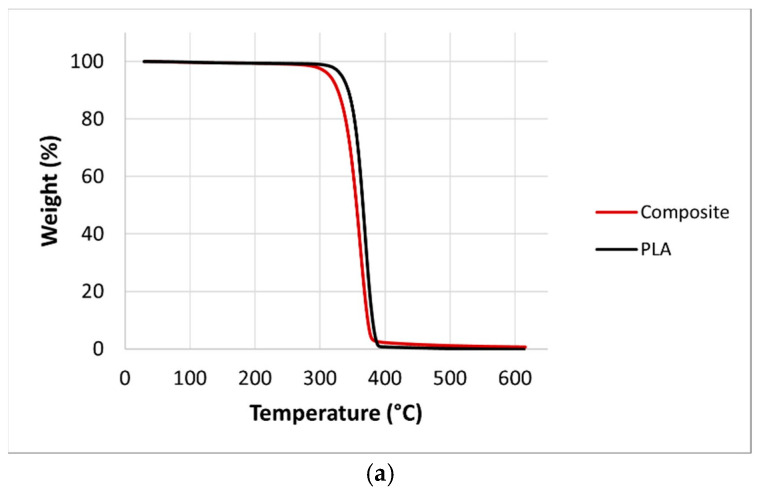

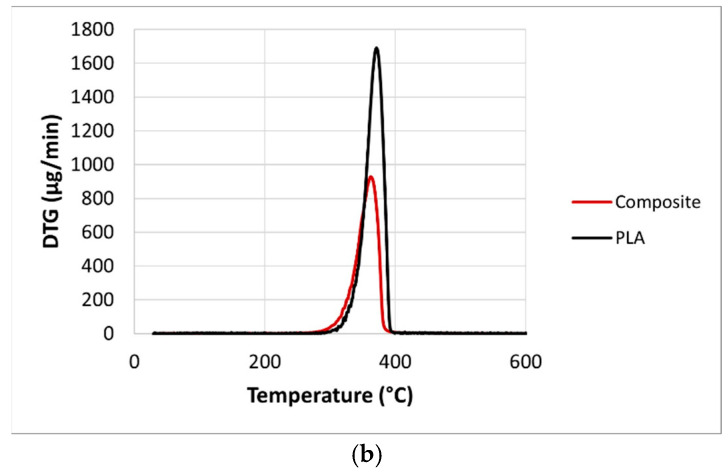
(**a**) TGA and (**b**) DTG curves of neat PLA and composite.

TGA and DTG curves of neat PLA and the continuous nettle fiber-reinforced PLA composite are shown in Figure 7. Analysis revealed that neat PLA exhibited the highest thermal stability, with T_onset_ and T_max_ values of 307 °C and 371 °C, respectively. In comparison, the PLA–nettle fiber composites showed lower values (T_onset_: 285 °C, T_max_: 362 °C), indicating a clear reduction in thermal resistance upon fiber addition. Nettle fibers alone presented even lower thermal stability, with T_onset_ and T_max_ of 269 °C and 353 °C, respectively. These findings demonstrate that the incorporation of nettle fibers, which are thermally less stable than PLA, leads to an earlier onset of degradation and overall deterioration in the thermal performance of the composites. Yussuf et al. (2010) indicated that replacement of polymer by less thermally stable material (natural fibers) results in a decreased thermal stability of the composite [44].

**Figure 8:**
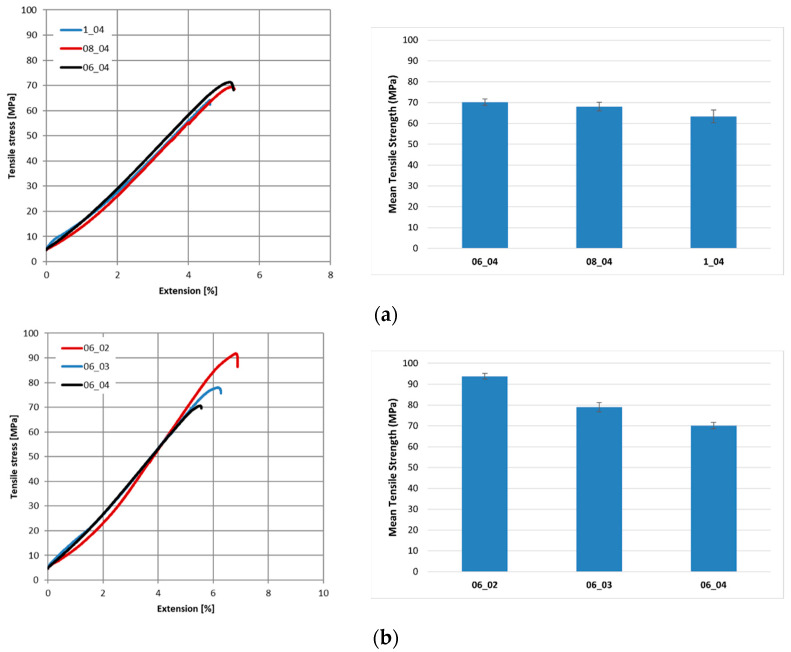
Representative tensile curves and bar chart of (**a**) 1_04, 08_04, 06_04; (**b**) 06_02, 06_03, 06_04.

Representative tensile curves of composites are shown in Figure 8a,b. A substantial improvement in tensile strength was also achieved through the reduction in hatch spacing, which complemented the effects of layer thickness variation. At a fixed layer thickness of 0.4 mm, narrowing the hatch spacing from 1 mm to 0.6 mm increased the tensile strength from 63.31 ± 3.04 MPa to 70.12 ± 1.49 MPa. This enhancement can be attributed to the greater overlap between adjacent deposition lines, which promotes structural continuity, reduces internal porosity, and strengthens the fiber–matrix interface. Improved interfacial bonding facilitates more efficient load transfer and enhances the overall mechanical stability of the composite. The literature supports this trend; Turkoglu et al. [42] and Bilir Kilinc et al. [41] reported that optimized line spacing resulted in superior stability and more uniform stress distribution for continuous fiber composites. Consistent with these findings, the present study confirms that narrow hatch spacing plays a decisive role in improving tensile strength and overall performance.

Similarly, decreasing the layer thickness led to a pronounced improvement in composite tensile strength. When the hatch spacing was held constant at 0.6 mm, reducing the layer thickness from 0.4 mm to 0.2 mm increased the tensile strength from 70.12 ± 1.49 MPa to 93.79 ± 1.37 MPa. This improvement is primarily linked to the enhanced interlayer adhesion enabled by thinner layers, which allows more effective fiber–matrix interaction and strengthens the mechanical integrity of the structure. Hou et al. [45] observed a comparable effect in continuous Kevlar fiber-reinforced PLA composites, where reduced layer thickness improved compressive strength by minimizing void content and increasing stress transfer efficiency. Similarly, Jiang et al. [46] and Bilir Kilinc et al. [43] reported that thinner layers enhance interlayer bonding and reduce porosity. Additionally, smaller layer thickness enables more precise fiber placement, facilitating a more uniform stress distribution and reducing localized stress concentrations. These observations reaffirm the critical role of layer thickness in improving composite performance, though it must be optimized in conjunction with hatch spacing for best results.

Table 5 presents the mechanical properties of nettle fiber-reinforced composites manufactured under different printing configurations. A clear trend can be identified, indicating that reductions in hatch spacing and layer thickness contribute positively to the mechanical performance of the composites. Specifically, these processing adjustments not only enhanced the elastic modulus but also promoted higher elongation at break, suggesting a more efficient stress transfer between the matrix and the reinforcing fibers. Such improvements highlight the strong influence of processing parameters on the overall structural integrity and ductility of the printed composites.

**Figure 9:**
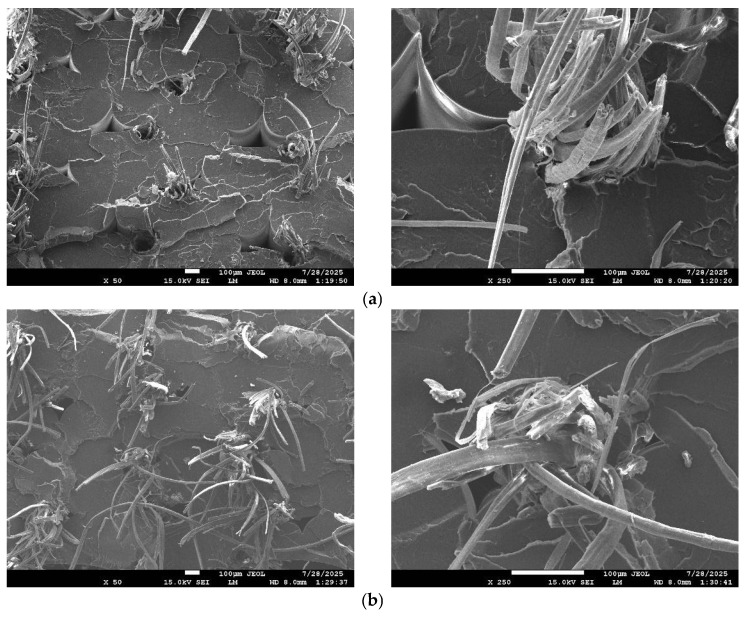

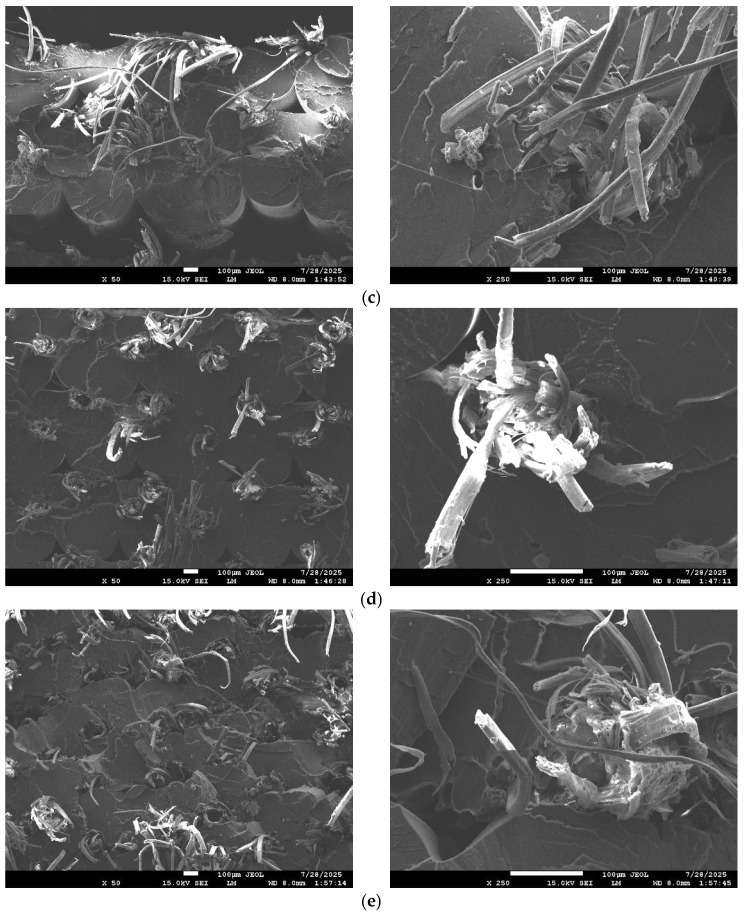
Fracture surface SEM images of (**a**) 1_04; (**b**) 08_04; (**c**) 06_04; (**d**) 06_03; (**e**) 06_02.

The post-tensile fracture surfaces of the composites, as observed through SEM, are presented in Figure 9. Regardless of the layer thickness and hatch spacing parameters, a common feature apparent across all images is the orderly and uniform deposition of fibers within the cross-sections. This indicates a consistent fiber alignment achieved during the printing process, which contributes to structural regularity. However, it is also evident that voids are present between adjacent deposited lines, originating from the layer-by-layer additive manufacturing process. When the layer thickness was fixed at 0.4 mm, a reduction in hatch spacing from 1.0 mm to 0.6 mm resulted in fibers being positioned more closely together, thereby increasing the reinforcement density within the composite. Similarly, with a constant hatch spacing of 0.6 mm, decreasing the layer thickness from 0.4 mm to 0.2 mm produced finer, more tightly stacked layers, enabling the fibers to be placed closer and thereby increasing the number of reinforcements per unit area. This closer fiber arrangement enhances the continuity of the composite structure, improves load bearing, and is consistent with the trends observed in the tensile strength results. These morphological findings suggest that both reduced hatch spacing and decreased layer thickness contribute synergistically to improved microstructural uniformity and mechanical performance. Thinner layers improve the contact surface between adjacent depositions, both of which facilitate more effective stress transfer throughout the composite. The observed fracture surface morphologies thus provide strong visual confirmation of the mechanical enhancements measured in tensile testing, highlighting the direct link between printing parameters, microstructural integrity, and performance outcomes.

## 4. Conclusions

This study demonstrated the fabrication of continuous nettle fiber-reinforced PLA composites using a custom-designed FDM-based 3D-printing system with in-nozzle fiber impregnation capability. The influence of two key printing parameters—hatch spacing and layer thickness—on fiber volume fraction, tensile performance, and fracture surface morphology was systematically examined.

The fiber volume fraction showed a clear dependence on printing parameters. At a fixed layer thickness of 0.4 mm, reducing the hatch spacing from 1.0 mm to 0.6 mm increased the fiber content from 7.94 vol.% to 12.21 vol.%. Similarly, when the hatch spacing was held constant at 0.6 mm, decreasing the layer thickness from 0.4 mm to 0.2 mm increased the fiber content from 12.21 vol.% to 24.43 vol.%.

Tensile test results revealed that decreasing hatch spacing from 1.0 mm to 0.6 mm at a fixed layer thickness of 0.4 mm improved tensile strength by 10.8% (from 63.31 MPa to 70.12 MPa). At a constant hatch spacing of 0.6 mm, reducing the layer thickness from 0.4 mm to 0.2 mm resulted in a greater improvement of 33.8% (from 70.12 MPa to 93.79 MPa). When compared to neat PLA, tensile strength was improved by 18.69% for the configuration of 1_04 and 75.83% for the configuration of 06_02. The maximum tensile strength achieved in this study was benchmarked against previously reported values for nettle fiber/PLA composites, with a comparative summary provided in Table 6. The findings clearly indicate that the application of 3D printing imparts a significant improvement in mechanical strength, underscoring its potential as an efficient processing route for natural fiber-reinforced polymer composites.

SEM analysis of post-tensile fracture surfaces showed that, regardless of parameter variation, fibers were deposited in an orderly and uniform manner. However, voids between deposited lines were present in all specimens. In regions subjected to tensile loading, both exposed fibers and voids were observed, indicating fiber pull-out due to interfacial debonding. Samples with reduced hatch spacing and thinner layer thickness exhibited closer fiber packing, consistent with the observed increases in tensile strength.

These findings confirm that changes in hatch spacing and layer thickness significantly influence the fiber content, microstructural features, and tensile behavior of continuous natural fiber-reinforced composites produced by FDM.

## Figures and Tables

**Table 1:** Chemical composition of nettle fibers and properties of PLA filament.

Chemical Composition of Fibers *	Properties of PLA Filament
Cellulose: 83.5%	Tensile strength: 53.34 MPa
Hemicellulose: 6.5%	Heat distortion temperature: 53 °C (0.45 MPa)
Lignin: 4.1%	Melt flow index: 5 (190 °C/2.16 kg)
Others: 5.9%	Density: 1.23 g/cm^3^

* Chemical composition of nettle fiber was based on the study of Bacci et al. (2009) [26].

**Table 2:** 3D-printing parameters.

3D-Printing Parameter	Unit
Printing temperature	210 °C
Printing speed	10 mm/s
Fiber orientation	0° (unidirectional)
Infill density	100%
Bed temperature	Room temperature
Hatch spacing	0.6 mm, 0.8 mm, 1 mm
Layer thickness	0.2 mm, 0.3 mm, 0.4 mm

**Table 3:** Labels of 3D-printed specimens.

Specimen Label	Hatch Spacing (mm)	Layer Thickness (mm)
1_04	0.4	1
08_04	0.4	0.8
06_04	0.4	0.6
06_03	0.3	0.6
06_02	0.2	0.6

**Table 4:** Fiber volume fractions of composites.

Specimen Label	Fiber Volume Fraction
1_04	7.94
08_04	9.92
06_04	12.21
06_03	16.29
06_02	24.43

**Table 5:** Mechanical properties of composites.

Specimen	Tensile Strength (Mpa)	Elastic Modulus (Gpa)	Elongation at Break (%)
06_02	93.79 ± 1.37	1.60 ± 0.05	6.87 ± 0.67
06_03	78.96 ± 2.21	1.45 ± 0.08	6.28 ± 0.84
06_04	70.12 ± 1.49	1.40 ± 0.08	4.7 ± 0.50
08_04	68.12 ± 4.08	1.39 ± 0.07	4.6 ± 0.47
1_04	63.31 ± 3.04	1.38 ± 0.04	4.4 ± 0.71

**Table 6:** Tensile strength comparison of nettle fiber-reinforced PLA composites.

Composite	Production Method	Orientation	Tensile Strength (Mpa)	Reference
Continuous nettle fiber–PLA	3D printing (FDM)	0°	93.79 ± 1.37	This work
Long nettle fiber (10 mm–70 mm)–PLA	Compression molding	0°	53.98 ± 3.11	[47]
Long nettle fiber (10 mm–70 mm)–PLA	Compression molding	45°	35.89 ± 2.58	[47]
Short nettle fiber (208.2 µm)–PLA	Melt compounding	Random	61.8 ± 0.5	[48]
Carded nettle fiber–PLA	Compression molding	Random (fiber web)	48.81 ± 10.2	[49]

## Data Availability

The original contributions presented in this study are included in the article. Further inquiries can be directed to the corresponding author.

## References

[1] Keskin O.Y., Koktas S., Seki Y., Dalmis R., Kilic G.B., Albayrak D. (2024). Natural cellulosic fiber from Carex panicea stem for polymer composites: Extraction and characterization. Biomass Convers. Biorefin..

[2] Dalmis R. (2023). Description of a new cellulosic natural fiber extracted from *Helianthus tuberosus* L. as a composite reinforcement material. Physiol. Plant..

[3] Viotti C., Albrecht K., Amaducci S., Bardos P., Bertheau C., Blaudez D., Bothe L., Cazaux D., Ferrarini A., Govilas J. (2022). Nettle, a long-known fiber plant with new perspectives. Materials.

[4] Dalmis R., Kilic G.B., Seki Y., Koktas S., Keskin O.Y. (2020). Characterization of a novel natural cellulosic fiber extracted from the stem of *Chrysanthemum morifolium*. Cellulose.

[5] Abdel Kader A.H., Fahmy T.Y., Kamel S. (2025). Lignocellulosic reinforced composites: A snapshot of progress. J. Wood Chem. Technol..

[6] Toplicean I.M., Ianus R.D., Datcu A.D. (2024). An overview on nettle studies, compounds, processing and the relation with circular bioeconomy. Plants.

[7] Bodros E., Baley C. (2008). Study of the tensile properties of stinging nettle fibres (*Urtica dioica*). Mater. Lett..

[8] Di Virgilio N., Papazoglou E.G., Jankauskiene Z., Di Lonardo S., Praczyk M., Wielgusz K. (2015). The potential of stinging nettle (*Urtica dioica* L.) as a crop with multiple uses. Ind. Crops Prod..

[9] Kılınç A.Ç. (2025). Characterization of novel fibers extracted from *Rumex obtusifolius* L. plant for potential composite applications. Mater. Test..

[10] Kılınç A.Ç., Köktaş S., Seki Y., Atagür M., Dalmış R., Erdoğan Ü.H., Göktaş A.A., Seydibeyoğlu M.Ö. (2018). Extraction and investigation of lightweight and porous natural fiber from *Conium maculatum* as a potential reinforcement for composite materials in transportation. Compos. Part B Eng..

[11] Pecas P., Carvalho H., Salman H., Leite M. (2018). Natural fibre composites and their applications: A review. J. Compos. Sci..

[12] Lotfi A., Li H., Dao D.V., Prusty G. (2021). Natural fiber–reinforced composites: A review on material, manufacturing, and machinability. J. Thermoplast. Compos. Mater..

[13] Sano Y., Matsuzaki R., Ueda M., Todoroki A., Hirano Y. (2018). 3D printing of discontinuous and continuous fibre composites using stereolithography. Addit. Manuf..

[14] Jamal M.A., Shah O.R., Ghafoor U., Qureshi Y., Bhutta M.R. (2024). Additive manufacturing of continuous fiber-reinforced polymer composites via fused deposition modelling: A comprehensive review. Polymers.

[15] Tian X., Todoroki A., Liu T., Wu L., Hou Z., Ueda M., Hirano Y., Matsuzaki R., Mizukami K., Iizuka K. (2022). 3D printing of continuous fiber reinforced polymer composites: Development, application, and prospective. Chin. J. Mech. Eng. Addit. Manuf. Front..

[16] Barbosa A.L., de Oliveira Romano R.C., Bernardes A.A., Gouvea D. (2025). Rheological and microstructural characterization of titania pastes for additive manufacturing using polymeric ceramic precursor as organic and inorganic binder. Ceram. Int..

[17] Turkoglu T. (2025). Experimental evaluation of biomimicry-inspired metallic hybrid lattice structures produced by laser powder bed fusion. Mater. Res. Express.

[18] Turkoglu T. (2024). Impact of lattice designs and production parameters on mechanical properties of AlSi10Mg in laser powder bed fusion. Mater. Test..

[19] Turkoglu T. (2024). Functional grading of polymer triply periodic minimal surface structures for enhanced compressive performance and lightweight design in additive manufacturing. J. Adv. Manuf. Eng..

[20] Guler S., Ozler B. (2025). Fabrication and analysis of Fe_3_O_4_/ZnO/epoxy composites via stereolithography: Structural and photocatalytic properties. Polym. Compos..

[21] Guler S., Yarali Cevik Z.B., Firat U., Demirgunes S.B., Ozler B. (2025). Investigation of titanium-reinforced polymer matrix nanocomposites fabricated by stereolithography for mechanical properties and activity in L929 cells. Rapid Prototyp. J..

[22] Kilinc A.C. (2025). Effect of line-width parameter on the tensile strength, surface profile and printing time of 3D printed PLA parts. Balikesir Univ. J. Sci. Eng..

[23] Karimi A., Rahmatabadi D., Baghani M. (2024). Various FDM mechanisms used in the fabrication of continuous-fiber reinforced composites: A review. Polymers.

[24] Khosravani M.R., Frohn-Sorensen P., Reuter J., Engel B., Reinicke T. (2022). Fracture studies of 3D-printed continuous glass fiber reinforced composites. Theor. Appl. Fract. Mech..

[25] Fatchurrohman N., Muhida R., Maidawati (2023). From corn to cassava: Unveiling PLA origins for sustainable 3D printing. J. Teknol..

[26] Bacci L., Baronti S., Predieri S., di Virgilio N. (2009). Fiber yield and quality of fiber nettle (*Urtica dioica* L.) cultivated in Italy. Ind. Crop. Prod..

[27] Tian X., Liu T., Yang C., Wang Q., Li D. (2016). Interface and performance of 3D printed continuous carbon fiber reinforced PLA composites. Compos. Part A Appl. Sci. Manuf..

[28] Ibrahim Y., Melenka G.W., Kempers R. (2018). Additive manufacturing of continuous wire polymer composites. Manuf. Lett..

[29] Waterbury M.C., Drzal L.T. (1989). Determination of fiber volume fractions by optical numeric volume fraction analysis. J. Reinf. Plast. Compos..

[30] Morales C.N., Claure G., Álvarez J., Nanni A. (2020). Evaluation of fiber content in GFRP bars using digital image processing. Compos. Part B Eng..

[31] Segal L., Creely J.J., Martin A.E., Conrad C.M. (1959). An empirical method for estimating the degree of crystallinity of native cellulose using the X-ray diffractometer. Text. Res. J..

[32] Canteri M.H., Renard C.M., Le Bourvellec C., Bureau S. (2019). ATR-FTIR spectroscopy to determine cell wall composition: Application on a large diversity of fruits and vegetables. Carbohydr. Polym..

[33] Titok V., Leontiev V., Yurenkova S., Nikitinskaya T., Barannikova T., Khotyleva L. (2010). Infrared spectroscopy of fiber flax. J. Nat. Fibers.

[34] Liang C.Y., Marchessault R.H. (1959). Infrared spectra of crystalline polysaccharides. II. Native celluloses in the region from 640 to 1700 cm.^−1^. J. Polym. Sci..

[35] Casoli A., Cremonesi P., Isca C., Groppetti R., Pini S., Senin N. (2013). Evaluation of the effect of cleaning on the morphological properties of ancient paper surface. Cellulose.

[36] Garside P., Wyeth P. (2003). Identification of cellulosic fibres by FTIR spectroscopy—Thread and single fibre analysis by attenuated total reflectance. Stud. Conserv..

[37] Geminiani L., Campione F.P., Corti C., Luraschi M., Motella S., Recchia S., Rampazzi L. (2022). Differentiating between natural and modified cellulosic fibres using ATR-FTIR spectroscopy. Heritage.

[38] Blackwell J., Vasko P.D., Koenig J.L. (1970). Infrared and Raman spectra of the cellulose from the cell wall of *Valonia ventricosa*. J. Appl. Phys..

[39] French A.D. (2014). Idealized powder diffraction patterns for cellulose polymorphs. Cellulose.

[40] Zhang B., Huang C., Zhao H., Wang J., Yin C., Zhang L., Zhao Y. (2019). Effects of cellulose nanocrystals and cellulose nanofibers on the structure and properties of polyhydroxybutyrate nanocomposites. Polymers.

[41] Kilinc F.B., Turkoglu T., Guler S., Kilinc A.C. (2025). Optimization of 3D printing parameters for enhanced tensile properties in continuous carbon fiber reinforced PLA composites. Mater. Res. Express.

[42] Turkoglu T., Kilinc A.C. (2025). Optimization of process parameters for steel wire-reinforced polylactic acid composites produced by additive manufacturing. Polymers.

[43] Kilinc F.B., Bozaci E., Kilinc A.C., Turkoglu T. (2025). Effect of atmospheric plasma treatment on mechanical properties of 3D-printed continuous aramid fiber/PLA composites. Polymers.

[44] Yussuf A.A., Massoumi I., Hassan A. (2010). Comparison of polylactic acid/kenaf and polylactic acid/rise husk composites: The influence of the natural fibers on the mechanical, thermal and biodegradability properties. J. Polym. Environ..

[45] Hou Z., Tian X., Zhang J., Li D. (2018). 3D printed continuous fibre reinforced composite corrugated structure. Compos. Struct..

[46] Jiang X., Shan Z., Zang Y., Liu F., Wu X., Zou A. (2024). Effect of process parameters on tensile properties of 3D printed continuous aramid fiber reinforced nylon 12 composites. J. Thermoplast. Compos. Mater..

[47] Bogard F., Bach T., Bogard V., Beaumont F., Murer S., Bliard C., Polidori G. (2022). Mechanical Properties of a PLA/Nettle Agro-Composite with 10% Oriented Fibers. Appl. Sci..

[48] Baumann J.R., Schönfeld L., Lühr C., Gusovius H.J., Akleh W., Govilas J., Placet V., Chalot M., Müssig J. (2025). Stinging nettle (*Urtica dioica* L.) as reinforcement in short fibre-reinforced biobased composites for application in injection moulding–A possible use case for biomass from marginal land?. Ind. Crops Prod..

[49] Dabi G.G., Wakjira Y.T., Feysa H.E., Abebe W.M. (2022). Development and characterization of laminated fiber reinforced bio-Composite From nettle and poly lactic acid fiber. J. Ind. Text..

